# Ultrasonography in trauma: a nation-wide cross-sectional investigation

**DOI:** 10.1186/s13089-017-0071-2

**Published:** 2017-06-20

**Authors:** Jesper Weile, Klaus Nielsen, Stine C. Primdahl, Christian A. Frederiksen, Christian B. Laursen, Erik Sloth, Hans Kirkegaard

**Affiliations:** 1 Emergency Department, Regional Hospital Herning, Herning, Denmark; 2 Research Center for Emergency Medicine, Aarhus University Hospital, Nørrebrogade 44, Building 1B, 8000 Aarhus C, Denmark; 3 Department of Medicine, Section of Respiratory Medicine, University Hospital Hvidovre, Hvidovre, Denmark; 4 Aarhus University, Aarhus, Denmark; 5 Department of Cardiology, Aarhus University Hospital, Aarhus, Denmark; 6 Department of Respiratory Medicine, Odense University Hospital, Odense, Denmark; 7 Department of Anesthesiology and Intensive Care, Aarhus University Hospital, Aarhus, Denmark; 8 Research Center for Emergency Medicine, Aarhus University Hospital, Aarhus, Denmark

**Keywords:** Trauma, Ultrasonography, Focused Assessment with Sonography in Trauma, FAST, Emergency Medicine

## Abstract

**Background:**

The Focused Assessment with Sonography in Trauma (FAST) protocol is considered beneficial in emergent evaluation of trauma patients with blunt or penetrating injury and has become integrated into the Advanced Trauma Life Support (ATLS) protocol. No guidelines exist as to the use of ultrasonography in trauma in Denmark. We aimed to determine the current use of ultrasonography for assessing trauma patients in Denmark.

**Methods:**

We conducted a nation-wide cross-sectional investigation of ultrasonography usage in trauma care. The first phase consisted of an Internet-based investigation of existing guidelines, and the second phase was a series of structured interviews of orthopedic surgeons, anesthesiologists, and radiologists on call in all hospitals receiving traumatized patients in Denmark.

**Results:**

Guidelines were obtained from all 22 hospitals receiving traumatized patients in Denmark. Twenty-one (95.5%) of the guidelines included and recommended FAST as part of trauma assessment. The recommended person to perform the examination was the radiologist in *n* = 11 (50.0%), the surgeon in *n* = 6 (27.3%), the anesthesiologist in *n* = 1 (4.5%), and unspecified in *n* = 3 (13.6%) facilities. FAST indications varied between circulatory instability *n* = 8 (36.4%), team leader’s discretion *n* = 6 (27.3%), abdominal trauma *n* = 3 (13.6%), and not specified *n* = 6 (27.3%). Telephone interviews revealed that exams were always *n* = 8 (36.4%) or often *n* = 4 (18.2%) registered in the patients’ charts. The remaining *n* = 10 (45.5%) facilities either never registered *n* = 2 (9.1%), it was not possible to register *n* = 1 (4.5%), or unknown by the trauma leaders *n* = 7 (31.8%). Images were often stored in *n* = 1 (4.5%), never stored in *n* = 10 (45.5%), not possible to store in *n* = 2 (9.1%), and unknown in *n* = 9 (40.9%) facilities.

**Conclusion:**

Ultrasonography was used in a non-uniform fashion by multiple specialties in Danish trauma facilities. Very few images from FAST examinations were stored and documentation was scanty. National guidelines on application and documentation of ultrasonography in trauma are called for.

**Electronic supplementary material:**

The online version of this article (doi:10.1186/s13089-017-0071-2) contains supplementary material, which is available to authorized users.

## Background

**Fig. 1:**
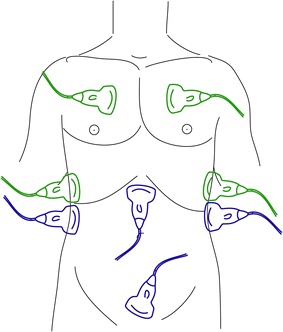
The FAST and eFAST exam. The* blue* probes illustrate the scanning positions of the FAST exam. The green probes illustrate the scanning positions of the extended FAST exam assessing for pneumothorax and hemothorax

The Focused Assessment with Sonography in Trauma (FAST) protocol is considered beneficial in emergent evaluation of trauma patients with blunt or penetrating injury [1]. FAST is a focused abdominal and cardiac ultrasound examination designed for assessment of free fluid in the peritoneum and the pericardium. The examination was initially introduced in the early 1990’s and has spread throughout the world [2]. The protocol includes three views of the abdomen and one view of the heart, as shown in Fig. 1. The abdominal views are right upper quadrant, left upper quadrant, and pelvis for assessment of hemorrhage into the peritoneal cavity. The subcostal view of the heart is used for detecting blood in the pericardium. The parasternal long axis view can be used as an alternative if the subcostal view cannot be obtained [3]. Previous studies of sensitivity and specificity of FAST have found these to be 63–100% and 95–100%, respectively [4]. The likelihood ratio of positive test has been found to be >7.9 [5]. The FAST exam has been shown to decrease time to intervention, complication rate, and hospital length of stay [6]. The FAST protocol is reproducible and not correlated to adverse effects from radiation[6].

In 2004, Kirkpatrick et al. presented an extension adding anterior and dorsolateral thoracic images of the pleura to the FAST exam [7]. See Fig. 1. The intention of the Extended Focused Assessment with Sonography in Trauma (eFAST) was to add detection of pneumothorax or hemothorax to the basic FAST. Ultrasonography has a higher sensitivity detecting pneumothorax than does antero-posterior chest X-ray in the supine position (88–98.1% vs. 50–75.5%) and can detect very small amounts of intrathoracic fluid [6–16].

In Denmark, there are no strict recommendations for trauma facilities to implement FAST, eFAST, or other ultrasound examinations as standard of care for traumatized patients. According to the Advanced Trauma Life Support (ATLS) protocol, FAST scanning should be performed as an extension to the primary survey [17], and the American College of Emergency Physicians (ACEP) recommends ultrasonography as initial diagnostic modality for blunt and penetrating abdominal trauma in hemodynamic unstable patients[18]. A previous Danish national study found that anesthesiologists or surgeons, present as consultants in the emergency department, perform ultrasonography in 43 and 18% of the cases, respectively [19]. It is unknown whether these are FAST examinations performed on trauma patients or other point-of-care examinations. Application and development of ultrasonography in trauma care are difficult if little is known about the existing usage of ultrasonography in the trauma facilities.

This study aims to clarify the current use of ultrasonography in existing trauma facilities, including examination indication, ultrasound protocol choice, examiner characteristics, and examination documentation.

## Methods

### Inclusion criteria

All 22 hospitals in Denmark receiving acutely injured patients via ambulance services (air, ship, or ground) with a predefined multidisciplinary trauma team, were included in this study. We defined the trauma patient as a patient activating the trauma call where a multidisciplinary trauma team cares for the patient in the trauma room.

We conducted a cross-sectional observational study performed in two phases.

#### Phase 1

- We gathered all local guidelines for trauma patient care from these 22 hospitals. This was done by searching publicly available resources on the Internet. If guidelines could not be accessed online, the Emergency Department was contacted by e-mail, and it sent the guideline.
- The guidelines from trauma facilities were investigated for use of ultrasonography in trauma. All mentions of “ultrasonography,” “ultrasound,” or “FAST” were recorded.

#### Phase 2

- The anesthesiologist, the orthopedic surgeon, and the radiologist on call in every trauma facility were interviewed via telephone by use of a structured questionnaire regarding the use of ultrasonography during treatment of traumatized patients. The questionnaire can be found in the Additional file 1: Appendix.
- All phone calls were conducted during weekdays between 09:00 am to 08:00 pm. A phone call was repeated seven times if not answered. After the 7th missed call, an e-mail containing the same questionnaire as the structured telephone interview was sent to the department. The department was asked to forward the questionnaire to a doctor frequently involved in trauma care. A second reminder e-mail was sent, and if the department did not respond, it was designated as “not responding.”

### Endpoints

Our primary endpoint was to establish the proportion of trauma facilities recommending ultrasonography in local trauma care manuals. Secondary endpoints were to establish the specialty of the physician conducting the FAST examination, the indication(s) for performing FAST, and whether other ultrasonography examinations were recommended in trauma care. Lastly, we examined the proportion of facilities documenting examinations in the patient records and the frequency of trauma facilities storing images from sonography.

### Statistics

Descriptive statistics was used to present actual numbers and percentages unless otherwise indicated in the text. We calculated Cohen’s weighted kappa for multiple observers on interview answers as to who was performing the examinations. When interpreting Cohen’s kappa, we used Landis and Koch’s guidelines from 1977 [20]. The interobserver agreement was also expressed in percentage of agreement. Calculations were performed using Stata 13 (Statacorp, USA).

## Results

Data were collected from August 2016 to December 2016. Trauma care manuals were obtained from all 22 facilities receiving traumatized patients in Denmark. A total of 64 (97.0%) out of 66 possible interviews were performed. Anesthesiologists, radiologists, and orthopedic surgeons on call on 22 hospitals were eligible for inclusion via telephone interviews. Anesthesiologists from all 22 hospitals participated in the study, while one eligible orthopedic department and one department of radiology failed to participate.

The FAST protocol was mentioned in 21 (95.5%) manuals for traumatized patients. One (4.5%) manual did not mention ultrasonography of any kind for trauma care.

**Fig. 2:**
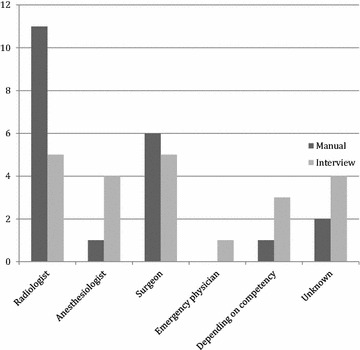
Specialties performing FAST. Specialties performing FAST examinations in trauma according to trauma care manuals and interviews with trauma leaders

The specialty of the performing physicians and the distribution of specialties are shown in Fig. 2. The results are from the 21 facilities mentioning ultrasonography in the trauma care manual, and from the interviews with trauma leaders from the 22 facilities. We interviewed orthopedic surgeons, anesthesiologists, and radiologists on call on the subject; Fig. 2 expresses the answer from the trauma leader. The numbers on the y axis are the number of hospitals. “Depending on competency” meant that the doctor present from either radiology, anesthesiology, or surgery with the highest level of competency performed the examination. In 8 (36.4%), all three interviewed physicians agreed on the person performing the FAST examination. In 6 (27.3%), two agreed, and in the remaining 8 (36.4%) there was total disagreement. Cohen’s kappa on interrater agreement among radiologists, anesthesiologists, and orthopedic surgeons was *K* = 0.35, *p* < 0.0001 and interpreted as fair.

**Fig. 3:**
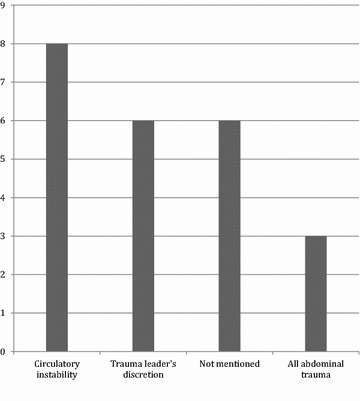
Indications (total *n* = 23) for performing FAST. Indications for performing FAST examination mentioned in the trauma manuals and number of hospitals

Indications for ultrasonography were either not mentioned, at the discretion of the trauma leader, abdominal trauma, or circulatory instability. The distribution is shown in Fig. 3. Because two trauma manuals stated indications as being abdominal trauma or circulatory instability, the total number of FAST indications (*n* = 23) was higher than the number of guidelines mentioning FAST (*n* = 21). The remaining facilities each stated a single indication as shown. One facility stated the indication as all circulatory unstable patients with suspected abdominal trauma; it was included as “circulatory instability” in the figure. In one manual, FAST exam was mentioned as a useful modality for “Trauma in Children.” As this indication was unspecific, it was not included in the figure. It was the only manual mentioning ultrasonography specifically for children. In 16 (72.7%) manuals, the FAST exam was part of the primary survey, and in 2 (9.1%), it was part of the secondary survey. The remaining 3 (13.6%) trauma facilities did not specify when the examination was to be performed.

The use of the eFAST protocol for ruling out pneumothorax and hemothorax was mentioned in 6 (27.3%) facilities. Additional uses for ultrasound examinations were mentioned in 7 (31.8%) trauma manuals, categorized as follows. Ultrasonography of arterial flow in extremities was mentioned in 5 (22.7%) manuals, but none of the manuals specify which specialty should perform the examination. Ultrasonography of scrotal trauma performed by radiologist was mentioned in two (9.1%) manuals, and ultrasonography of internal organs by radiologist was mentioned in two (9.1%) manuals. One (4.5%) manual mentioned focused cardiac ultrasonography in the primary survey, but it did not mention indication or who was to perform the examination. Lastly, one (4.5%) manual mentioned unspecified thoracic ultrasonography for penetrating trauma.

**Fig. 4:**
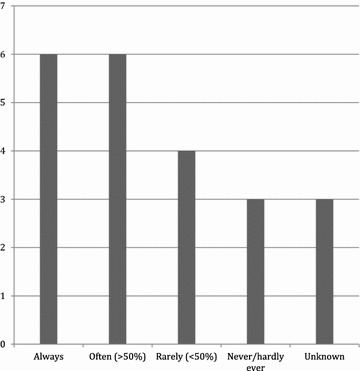
Ultrasonography usage according to trauma leaders. Usage according to trauma leaders when asked how frequently ultrasonography is used in trauma care in their facility and number of hospitals

Trauma leaders were questioned in regards to the frequency of use of ultrasonography in trauma care. Answers are shown in Fig. 4.

**Fig. 5:**
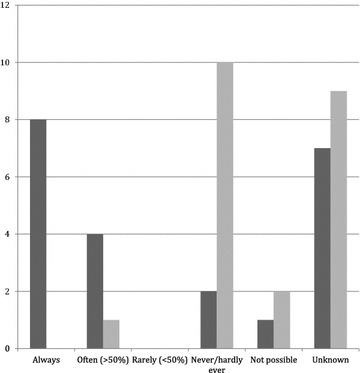
Documentation. Documentation in patient charts and storage of ultrasonography images when ultrasonography is performed. Results from interviews with trauma leaders in all facilities. The *graph* shows the distribution of answers to two questions. *Dark gray*: “Are the results of the ultrasonography examination documented in the patient chart?” *Light gray*: “Are the images stored?”

Lastly, trauma leaders were questioned about the frequency of storing images and entering descriptions of the ultrasound examinations into medical charts. Results are shown in Fig. 5.

## Discussion

The FAST exam was recommended in 21 out of 22 local guidelines. In addition, our study revealed three main findings. First, different specialties seem to perform ultrasound examinations in different trauma facilities. Second, we found various indications for performing FAST, ranging from all “abdominal trauma” to “trauma leader’s discretion.” Third, we found that documentation of ultrasonography examinations and storing of images was sparse.

An e-mail survey from 2008 in the U.S. showed that 85% of hospitals reported using the FAST examination for trauma care [2]. Our investigation showed a recommendation of FAST in 95.5% of all facilities receiving traumatized patients in Denmark. The higher percentage in our study probably reflects the growing interest in ultrasonography over time. Repetition of the American study might reveal a higher percentage today, which is why we consider the difference as a reflection of general development in the area.

Our study revealed differing local recommendations regarding which specialties should perform the ultrasound examinations. We have not come across evidence in the literature that one specialty should outperform others. We found that fewer surgeons and radiologists, and more anesthesiologists, perform FAST examination according to interviews than according to the local trauma care guidelines. Earlier work has shown that the vast majority of physicians performing ultrasonography in the Emergency Department are anesthesiologists [19]. Our study suggests that in trauma it will more often be the person with the greatest skill who performs the FAST examination rather than the person recommended in the guidelines. These findings might reflect that anesthesiology often is the specialty present with the most experience, while radiologists may be on call but not present in the trauma room.

The FAST exam is user dependent, and the specificity and sensitivity are related to the experience of the provider. The examination has low sensitivity for organ injury without hemoperitoneum and low sensitivity for retroperitoneal bleeding [6]. To reach an overall sensitivity of 67% and specificity of 100%, extensive experience of more than 100 FAST examinations is necessary [21]. A reliable and validated assessment tool for assessment of competency in point-of-care ultrasonography exists [22]. This evidence calls for a specification in the existing guidelines recommending the most experienced person to perform the examination. Preferably, the experience should be quantified, and an expected minimum of experience or a minimum required competency level should be outlined.

The indications for performing FAST differ between facilities, and, including extended FAST, views of the thorax are also heterogenic. Some facilities recommend other use of sonography beyond FAST, such as sonography of the scrotum. The addition of extended FAST views appears helpful in the literature as life-threatening injuries such as tension pneumothorax or massive hemothorax can be diagnosed at the bedside [11, 23]. Cardiac evaluation alone in penetrating trauma to the chest has been shown to decrease mortality [24]. Although the eFAST exam for trauma has not been shown to reduce mortality, it still has several advantages. Knowledge of thoracic injury with pneumothorax, hemothorax, or pericardial effusion at the time of arrival can be used to guide initial treatment even before a CT scan is performed. Future trials should seek to illuminate the clinical strength of the full eFAST over the FAST alone.

To our knowledge, the frequency of ultrasonography images being stored and documented has not previously been investigated in the trauma setting. Recommendations have been published on how to store images and document examinations [25], but these are not followed. Our findings, which show a surprisingly low frequency of storage and documentation, call for attention to the problem in the local guidelines. Without proper documentation, developing the field is immensely difficult. Furthermore, lack of image storing may present legal issues in the future.

Our study has limitations. First, the study is national and limited to the Danish hospitals receiving traumatized patients. However, our findings of particular problems in heterogeneity between facilities and scanty documentation undoubtfully exist abroad, and our call for national guidelines can only inspire other countries to do the same. A second limitation is ascribing value to interviews of the trauma team leaders and other doctors on call. We do not know whether the results would have been different if the phone call had been made on a different day or whether the answers were influenced by recollection bias.

## Conclusion

Ultrasonography is applied in a heterogeneous manner by multiple specialties on multiple indications in trauma care in Denmark. Storage and documentation of examinations is sparse and desultory. Multispecialty national evidence-based guidelines, as well as unified implementation of existing guidelines, are called for.

## Additional file

**Additional file 1.** Appendix 1

## Abbreviations

ATLS: Advanced Trauma Life Support
eFAST: extended Focused Assessment with Sonography in Trauma
FAST: Focused Assessment with Sonography in Trauma
