# Supplementary material for: Ultrasonography in trauma: a nation-wide cross-sectional investigation
Source: Crit Ultrasound J. 2017 Jun 20;9:16. doi: 10.1186/s13089-017-0071-2 (PMC5479771; doi:10.1186/s13089-017-0071-2)
Supplement: Supplementary file 1 — Additional file 1. Appendix 1 [file 13089_2017_71_MOESM1_ESM.docx]

**APPENDIX for: Ultrasonography in Trauma – A national cross sectional investigation**

**1. Interview guide // Ultrasonography in trauma**

Questionnaire for the trauma team leader

Date: ____ / ____ / _____, Time: _____ : _____ / Investigator: ______________________________

Hospital: _____________________________ / Department: ________________________________

🞐: Gives oral informed consent to participate in the study

Name: _____________________________________

Is ultrasonography used in trauma care?:

🞐: Always 🞐: Often (>50%) 🞐: Rarely (<50%) 🞐: Never/Hardly ever 🞐: Unknown

Who performs the ultrasound examinations, if they are performed?:

🞐: Radiologist 🞐: Anesthesiologist 🞐: Surgeon 🞐: Emergency 🞐: Other: __________________

Which modality is performed for trauma?:

🞐: FAST 🞐: eFAST 🞐: Focused Cardiac ultrasound 🞐: Vascular Access

🞐: Focused ultrasound of the lungs 🞐: Other: _______________________________________

Are the results of the ultrasonography examination documented in the patient chart?:

🞐: Always 🞐: Often (>50%) 🞐: Rarely (<50%) 🞐: Never/Hardly ever 🞐: Unknown

Are the images stored?

🞐: Always 🞐: Often (>50%) 🞐: Rarely (<50%) 🞐: Never/Hardly ever 🞐: Unknown

Only if special pathology is found: ________________________________________

Where are the images stored?:

🞐 Locally on the US machine 🞐 Server 🞐 In the patient chart 🞐 Other: _______________
